# Genotyping-by-sequencing in an orphan plant species *Physocarpus opulifolius* helps identify the evolutionary origins of the genus *Prunus*

**DOI:** 10.1186/s13104-016-2069-4

**Published:** 2016-05-11

**Authors:** Matteo Buti, Daniel J. Sargent, Khethani G. Mhelembe, Pietro Delfino, Kenneth R. Tobutt, Riccardo Velasco

**Affiliations:** Research and Innovation Centre, Fondazione Edmund Mach, via Mach 1, 38010 San Michele All’adige, TN Italy; ARC Infruitec-Nietvoorbij, Private Bag X5026, Stellenbosch, 7599 South Africa; Driscoll’s Genetics Ltd., East Malling Enterprise Centre, New Road, East Malling, Kent, ME19 6BJ UK

**Keywords:** Genome evolution, Comparative mapping, Solanaceae, Genomics, Plant breeding

## Abstract

**Background:**

The Rosaceae family encompasses numerous genera exhibiting morphological diversification in fruit types and plant habit as well as a wide variety of chromosome numbers. Comparative genomics between various Rosaceous genera has led to the hypothesis that the ancestral genome of the family contained nine chromosomes, however, the synteny studies performed in the Rosaceae to date encompass species with base chromosome numbers *x* = 7 (*Fragaria*), *x* = 8 (*Prunus*), and *x* = 17 (*Malus*), and no study has included species from one of the many Rosaceous genera containing a base chromosome number of *x* = 9.

**Results:**

A genetic linkage map of the species *Physocarpus opulifolius* (*x* = 9) was populated with sequence characterised SNP markers using genotyping by sequencing. This allowed for the first time, the extent of the genome diversification of a Rosaceous genus with a base chromosome number of *x* = 9 to be performed. Orthologous loci distributed throughout the nine chromosomes of *Physocarpus* and the eight chromosomes of *Prunus* were identified which permitted a meaningful comparison of the genomes of these two genera to be made.

**Conclusions:**

The study revealed a high level of macro-synteny between the two genomes, and relatively few chromosomal rearrangements, as has been observed in studies of other Rosaceous genomes, lending further support for a relatively simple model of genomic evolution in Rosaceae.

**Electronic supplementary material:**

The online version of this article (doi:10.1186/s13104-016-2069-4) contains supplementary material, which is available to authorized users.

## Background

The Rosaceae is a large and diverse family of around 90 genera containing over 3000 species that encompass many fruit species. These include apples, cherries, raspberries and strawberries, along with ornamental species, such as rose, and some timber species. There exists remarkable morphological divergence between genera and species within the family [1] including: a variety of fruit types, such as pomes, drupes and achenes; diversity in plant habit, including herbs, shrubs and trees; and variation in chromosome number, from *x* = 7 in *Fragaria, Rubus, Rosa* and related genera, to *x* = 17 in *Malus*, *Pyrus* and related genera. In the phylogeny proposed by Potter et al. [1], the family was divided into three sub-families, the Rosoideae, the Dryadoideae and the Spiraeoideae, with the Spiraeoideae containing seven tribes encompassing a wealth of chromosomal diversity including *n* = 8 (Amygdaleae), *n* = 9 (Neillieae) and *n* = 17 (Pyreae).

Comparative genomic studies between species and genera of the Rosaceae have been performed in order to assess the possibility of extrapolating genomics information from one species to assist in understanding genetic processes in others. Early studies between species of different genera used conserved orthologous RFLP markers to investigate the synteny between linkage groups of two genera now both assigned to the Spiraeaoideae, *Malus* (Pyreae 2*n* = 34) and *Prunus* (Amygdaleae 2*n* = 16) [2]; they identified several examples of homology between one *Prunus* linkage group (LG) and two *Malus* LGs and also demonstrated evidence for a fusion-fission event on the large LG1 of *Prunus* and the non-homologous LG13 and LG8 in *Malus*. Later, through the comparison of linkage maps of *Prunus* (Amygdaleae) and *Fragaria* (Rosoideae 2*n* = 14) using both RFLP and PCR-based markers, genome wide macro-synteny was evaluated across two sub-families [3]. A total of 71 markers, comprising 40 RFLPs and 31 EST/gene-specific markers, were mapped in *Prunus* and *Fragaria* and revealed a high degree of synteny between the linkage maps, with most markers that mapped to a single LG in one species mapping to one or two LGs in the other. Vilanova et al. [3] identified sufficient structural conservation between the genomes of *Fragaria* and *Prunus* for an ancestral genome configuration for the Rosaceae containing nine chromosomes to be proposed. A total of 36 chromosomal rearrangements were required to reconstruct the ancestral genome, with an estimated time from divergence from a common ancestor of ~29 million years [3]. The subsequent release of genome sequences for three Rosaceous species, in *Fragaria*, *Malus*, and *Prunus* [4–6] permitted synteny studies to be performed at higher resolution and with greater precision than had been possible using linkage mapping alone. While these studies supported the hypothesis of an ancestral genome containing nine chromosomes they also provided further insights into the mechanisms that have shaped the evolution of the genomes of the various genera within the family that encompass such diversity in traits important to man [7, 8]. Whilst the ancestral genome of the Rosaceae has been hypothesized to contain nine chromosomes, the synteny studies performed in the Rosaceae to date encompass species with base chromosome numbers *x* = 7 (*Fragaria*), *x* = 8 (*Prunus*), and *x* = 17 (*Malus*), and no study has included species from one of the many Rosaceous genera containing a base chromosome number of *x* = 9.

The ornamental genus *Physocarpus* (Nelliaea 2*n* = 2*x* = 18) in the Spiraeoideae was positioned as an immediate sister genus to *Prunus* in a comprehensive phylogeny of the Rosaceae [1]. A molecular map, based on a segregating F_2_ progeny was reported for the species, which spanned the expected nine linkage groups and contained a total of 181 molecular markers across 586.1 cM, along with two genes controlling leaf colour [9]. Although the authors reported the positions of three sequence-characterised gene-specific markers and a single *Malus* SSR marker, the remaining 177 markers were either AFLP or RAPD markers and thus were not readily applicable to comparative genomic studies with other Rosaceous genera.

Until recently, the development of molecular resources in ‘orphan’ species was time consuming and expensive since it involved the development and sequencing of enriched genomic libraries to produce species-specific or genus-specific tools such as microsatellites [10], or the identification of polymorphisms in conserved orthologous sequences, where sequence variability is low [11]. With the advent of second-generation sequencing technologies however, techniques such as genotyping by sequencing (GBS) [12] and related genotyping methodologies permit the rapid development of an abundance of segregating molecular markers, without the need for any a priori knowledge of the structure of the genome of the organism under investigation. The GBS method has been applied to a diverse range of organisms, including members of the Rosaceae such as *Fragaria* [13], *Rubus* [14] and *Malus* [15].

In this investigation, we have elaborated the previously published genetic linkage map of *Physocarpus* [9] using SNP markers identified through GBS. We used this map to study the extent of the genome diversification between the *Physocarpus* genus, with a base chromosome number of *x* = 9 and *Prunus*, *x* = 8, its phylogenetically close relation for which a full genome sequence is available. The results of the study illuminate the nine chromosome ancestral model previously reported for the Rosaceae [3, 4, 7, 8].

## Methods

### *Physocarpus* mapping population and DNA extraction

A segregating progeny (Phy-5) derived from a sib-cross of two seedlings from the cross *P. opulifolius* ‘Diabolo’ × ‘Luteus’ (and thus approximating to an F_2_ population) was previously raised for the purposes of mapping leaf colour characteristics [9]. DNA from the 94 individuals of the population and the two parental lines (‘764-3′ and ‘764-Z’) was extracted using the DNeasy plant mini kit (Qiagen) following the manufacturers’ recommendations and was diluted to 10 ng/µl for analysis using genotyping-by-sequencing (GBS).

### GBS, data analysis and marker identification

Genotyping was performed with the adaptors and protocols suggested by Elshire et al. [12] using the *ApeKI* restriction enzyme and adaptor dilutions as described by Ward et al. [14]. Briefly, 100 ng of DNA from each of the parents and 94 seedlings were digested with 3.6 U of *Ape*KI and subsequently, 1.8 ng of the uniquely barcoded adaptors was ligated using T4 DNA ligase (New England Biolabs). Reactions for each individual genotype were performed separately with a unique adaptor, following which all samples were pooled and a PCR amplification was performed on the pooled library. The pooled library was purified using a QiaQuick PCR purification column according to the manufacturers’ protocol and the purified library was sequenced on a single lane of a HiSeq 2000 sequencing platform flow cell (Illumina, San Diego, USA) using 101 single-end cycles.

Samples were initially de-multiplexed using custom perl scripts reported by Elshire et al. [12] and retrieved from the GBS barcode splitter site on sourceforge [16]. Subsequently, data were analysed using STACKS v1.29 [17] running *stacks denovo* with default settings. The resultant genotype files were filtered for those individuals containing more than 50 % missing data, and subsequently those loci containing more than 50 % missing data. The tags for the genotypes that remained were used as queries for BLAST. BLASTN v2.2.28+ [18] was used running default parameters against the published hardmasked *Prunus persica* v1.0 genome sequence [6] and those loci that gave an unambiguous match, i.e., mapping to a unique site on the *P.persica* genome with greater than 90 % sequence identity and a cut off E-value of 1e-15, were retained for further analysis. Since the Phy-5 mapping population approximates to an F_2_ progeny, to simplify the mapping process, only SNP markers heterozygous for the same two alleles in both parental lines (i.e., AB × AB) were retained for segregation analysis and linkage map construction.

### Linkage map construction and analysis of synteny

The segregating marker dataset of Sutherland et al. [9] was combined with the markers identified through GBS following the criteria listed above for linkage map construction using JOINMAP 4.1 (Kyazma, NL). Linkage map construction essentially followed the procedures using regression mapping reported previously [9] except that the latest version of JOINMAP (v4.1) was employed. The mapping data obtained were visually inspected to eliminate any spurious genotype calls in the GBS data that created unlikely double recombination events. Any unlikely GBS genotype calls were converted to missing values. Synteny was investigated by comparing the map positions of all GBS markers to the corresponding location of their sequence tags in the *P. persica* genome sequence obtained following the BLAST analysis described above. The relative positions of the markers were visualized by plotting the Phy-5 linkage groups against the *Prunus* pseudomolecules (the first eight contigs in the assembly) using Circos v0.67 [19]. Inversion, translocation and fusion-fission events since the divergence of the species from a common ancestor were inferred and a model of subsequent genome evolution was proposed on the basis of the discrete syntenic blocks observed to be conserved between the two genomes following the criteria detailed in [7]. Thus a syntenic block on the *Physocarpus* linkage map was defined if it contained a minimum of three sequential SNP markers located within 3.5 Mbp of each other on the *Prunus* genome. *Physocarpus* linkage groups were relabelled where possible according to their relationships with the *P. persica* pseudomolecules and the degree of similarity shared between the two genomes.

## Results

### GBS and BLAST analysis

A total of 94,558,351 reads were produced from sequencing of the GBS library and the average number of reads per genotype used in map construction was 1,170,375. Following analysis with STACKS, 15,908 segregating SNP markers were identified from the GBS library developed for *Physocarpus*. A total of 62 seedlings contained data for at least 50 % of the segregating SNPs identified, whilst the remaining seedlings were omitted from further analysis. Subsequently, a total of 8730 segregating SNP loci contained data for at least 50 % of the 62 seedlings and, when the tag sequences from these SNPs were used as queries to BLAST the published *P. persica* v1.0 genome sequence, a total of 255 tags significantly matched a single unambiguous *Prunus* locus. These loci were retained for linkage map construction along with data from the previously published Phy-5 linkage map [9].

### Linkage map development

**Fig. 1:**
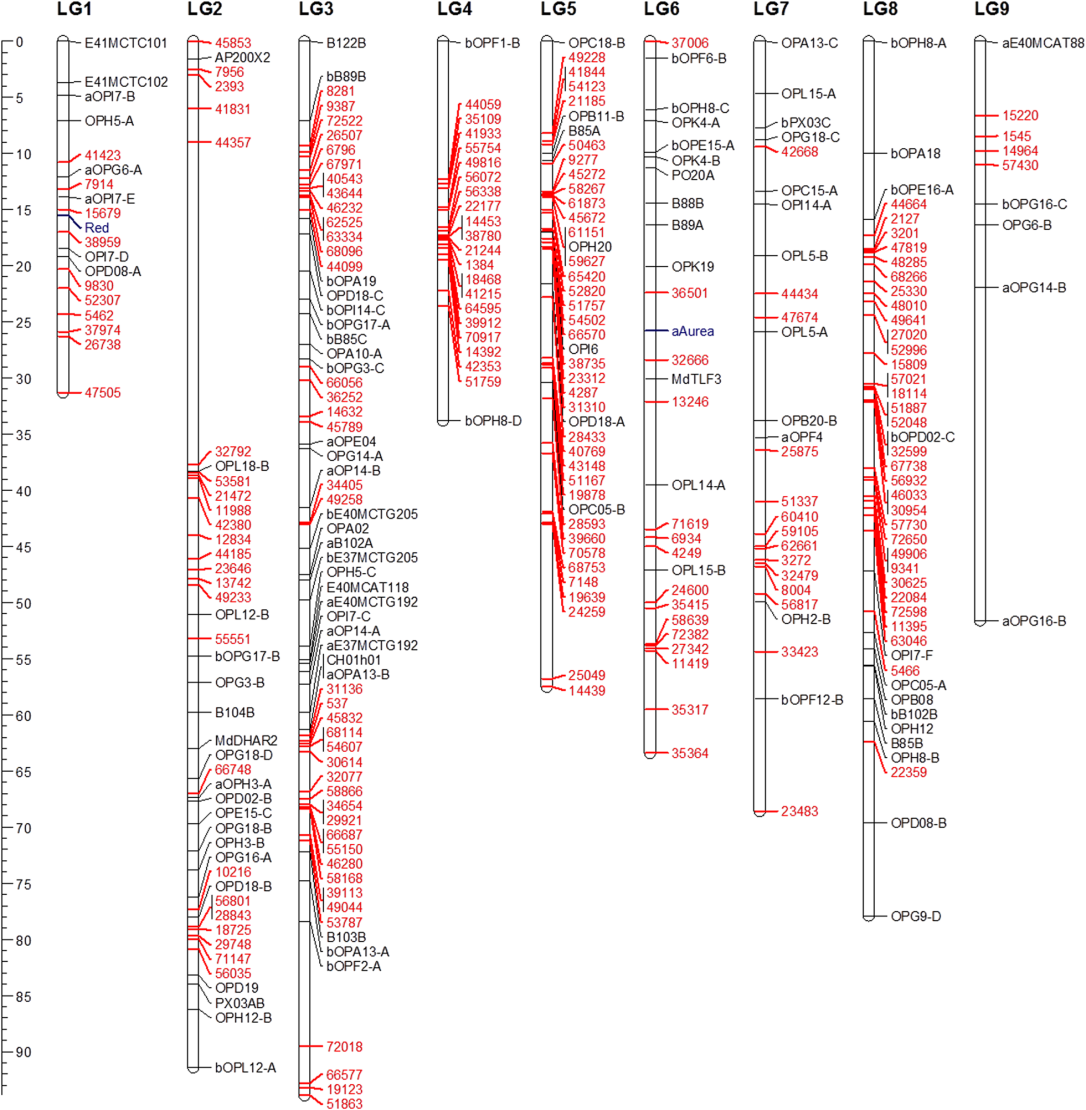
*Physocarpus opulifolius* linkage map. A genetic linkage map of an F_1_ *P. opulifolius* mapping population containing 222 GBS-derived SNP markers, along with 96 RAPDs, nine AFLPs, four gene specific markers, one SSR and two phenotypic traits mapped previously, spanning a total of 413.7 cM across nine linkage groups Newly mapped SNP markers are given in *red*, whilst phenotypic markers are given in *blue*. The nine linkage groups are labelled LG1 to LG9 according to their relationships with *Prunus persica* linkage groups

Data for the previously published molecular markers and phenotypic traits for the Phy-5 mapping progeny were combined with data for the 255 SNP markers produced using GBS for 62 seedlings of the mapping population. The linkage map produced contained the expected nine linkage groups associated with the *Physocarpus* chromosomes. The linkage groups contained a total of 332 molecular markers—222 SNP markers developed through GBS, 96 RAPDs, nine AFLPs, four gene specific markers and one SSR—and the two phenotypic traits mapped previously and the map spanned a total of 413.7 cM. All linkage groups contained newly mapped GBS SNP markers (Fig. 1), LG3 containing the most SNP markers (47) and LG9 the least (4) (Table 1). Of the two genes controlling phenotypic traits for leaf colour, *Aur* mapped to LG6 and was flanked by two RAPD/AFLP markers originally reported by Sutherland et al. [9] while *Pur* mapped to LG1 and was flanked by two SNP markers revealed through GBS analysis with orthologous loci on the *Prunus* genome sequence (Fig. 1).

**Table 1:** Summary statistics for the SNP-based linkage map of *Physocarpus opulifolius* detailing the linkage group lengths in cM, and the number of SNP, RAPD, AFLP, gene-specific, SSR and phenotypic markers mapping to each linkage group

LG	Length	SNP	RAPD	AFLP	Gene	SSR	Phenotype
LG1	28.862	13	6	2	1	0	1
LG2	70.099	29	17	0	2	0	0
LG3	39.207	47	21	5	0	1	0
LG4	34.267	20	2	0	0	0	0
LG5	57.085	41	9	1	0	0	0
LG6	40.045	22	11	0	1	0	1
LG7	44.763	14	12	0	0	0	0
LG8	47.758	32	14	0	0	0	0
LG9	51.576	4	4	1	0	0	0
Total	413.66	222	96	9	4	1	2

### Comparative analyses of *Physocarpus* and *Prunus* genomes

**Fig. 2:**
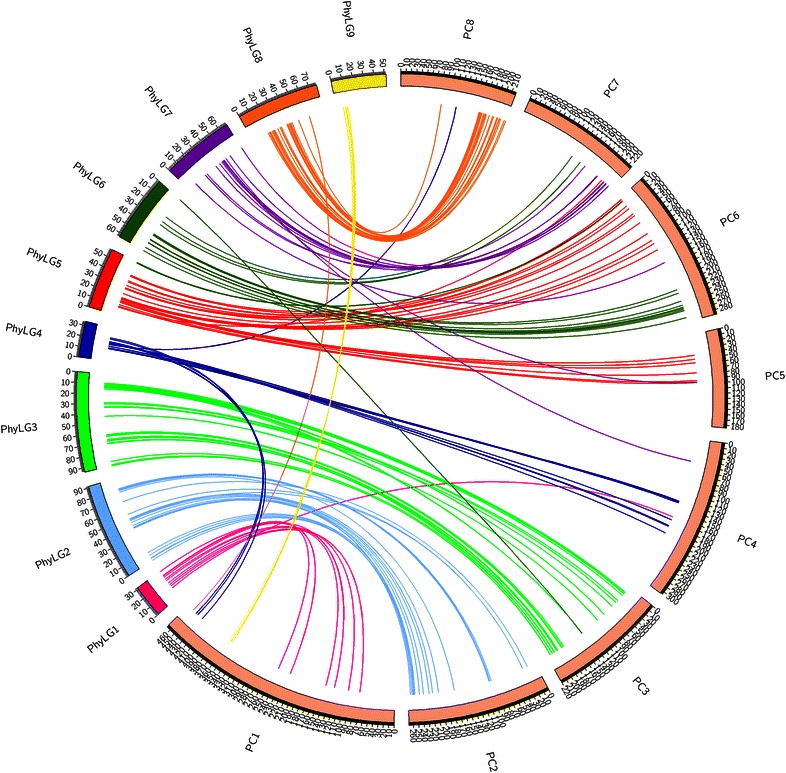
Orthology map of *Prunus persica and Physocarpus opulifolius.* An orthology map of the relationships between the eight pseudomolecules of the *P. persica* genome sequence and orthologous markers mapped to the nine linkage groups of the *P. opulifolius* linkage map. *Physocarpus* linkage groups are shown on the *left* of the figure and genetic distances are given in cM, whilst *Prunus* pseudomolecules are given to the *right* and their physical distances are given in 100,000 bp intervals. Links between linkage groups and pseudomolecules indicate the positions of orthologous markers on the two genomes

The positions of the 222 SNPs distributed throughout the nine linkage groups of *Physocarpus* were compared to their positions on the eight pseudomolecules of *Prunus* (Fig. 2; Additional file 1: Figures S1–S9). Figure 2 depicts all marker positions, including markers not contained in syntenic blocks, whilst Additional file 1: Figures S1–S9 show only those markers that identify chromosome scale syntenic relationships. *Physocarpus* linkage groups LG1, LG2, LG3, LG6, LG7, LG8 and LG9 contained the majority of markers in syntenic blocks that were syntenic with just a single *Prunus* chromosome each according to the analysis criteria followed, whereas groups LG4 and LG5 contained syntenic blocks located on two *Prunus* chromosomes. Close scrutiny of marker positions on the Phy-5 linkage map of the orthologous SNP sequences permitted the identification of a set of conserved syntenic blocks between *Physocarpus* and *Prunus* as follows.

LG1 of the Phy5 map was syntenic with PC1, with the majority of markers (77 % of those mapped) displaying a high degree of synteny (Additional file 1: Figure S1). LG2 was syntenic with PC2, with the majority of markers (89.7 %) displaying a high degree of synteny (Additional file 1: Figure S2) and just three markers whose positions suggested a possible inversion event towards the proximal end of the LG/PC. LG 3 displayed a high degree of colinearity and thus synteny with PC3, with just four markers (8.5 %) locating to non-colinear positions (Additional file 1: Figure S3). The analysis of markers located on LG4 showed their positions were syntenic with a large section of PC4, and a smaller section of PC1, revealing a major fusion-fission event between these chromosomes (Additional file 1: Figure S4). Likewise LG5 was syntenic with PC6 at the proximal end and with PC5 at the distal end revealing a further fusion-fission event (Additional file 1: Figure S6). LG 6 was completely colinear with the distal-most 6 Mb of PC6, and likewise LG7 and LG8 were highly syntenic and almost completely colinear with the distal sections of PC7 and PC8 respectively (Additional file 1: Figures S6–S8). Finally, markers mapped to LG9 were syntenic with a small (1 Mb) section of PC1, indicating a probable further fusion-fission event between the genomes of the two genera (Additional file 1: Figure S9). Additional markers not considered to be part of clearly defined syntenic blocks were present on seven linkage groups and their positions are detailed in Additional file 2: Table S1.

## Discussion

### Linkage map construction using genotyping by sequencing

In this investigation, the Phy5 linkage map reported by Sutherland et al. in [9], which was composed primarily of sequence uncharacterized AFLP and RAPD markers, was elaborated using SNP markers of defined sequence, developed using GBS. The GBS approach, first described in 2011 by Elshire et al. [12], is a method for the development of near saturated linkage maps of any given progeny for which there exists allelic segregation and for which DNA is available for library construction. The approach had been successfully applied to a range of plant species including barley and wheat [20] and the Rosaceous species *Rubus idaeus* and *Malus pumila* [14, 15], providing significant insights into the genomes of those species and the genetics of important morphological traits. Since no prior information about the genomes of the progeny under investigation is required for marker identification and scoring, it is an ideal approach to characterize the genomes of orphan crop species rapidly and cost effectively and to provide sequence data about the loci mapped.

In the Phy5 mapping population examined here, a potential SNP-set was identified containing a total of 15,908 polymorphic tag sites. However, since the aim of this investigation was to compare genomic arrangements between the Phy5 linkage map and the *Prunus* genome, and to provide an extra layer of information regarding the SNPs identified and mapped in the Phy5 progeny, only the 222 (1.4 %) SNPs that identified reliable orthologous sites on the *Prunus* genome were carried forward for linkage map construction. Despite *Prunus* being the closest sister genus to *Physocarpus* in the phylogeny of the Rosaceae [1], this mapping criterion significantly reduced the number of markers which were available for mapping. Since sequence tags often contained SNPs at the ends of the stacks from which they were developed (data not shown), the tag sequences alone are insufficient for subsequent marker development, and thus additional reference sequence is often, but not always, necessary for downstream application or for transfer of the SNPs identified to other genotypes. This point highlights a major weakness of performing linkage mapping using GBS with no additional sequence information for the species under investigation. Depending on the genetic distance between orphan species investigated and their better characterized cousins, reliance on the identification of orthologous sequence information from sister taxa limits the resolving power of any investigations performed.

However, the advent of recent iterations of second generation sequencing platforms with greater read-length capabilities raises the possibility of significantly increasing the resolution of such studies through low-coverage sequencing of mapping population parents. Illumina MiSeq represents a cost-effective platform for the generation of relatively long reads which, combined with judicious library insert choice and the use of methodologies such as ‘flashing’ of overlapping paired reads [21] followed by subsequent assembly, would provide a basic, yet highly informative ‘reference’ genome sequence to which identified tag sequences could be associated through BLAST analysis. This approach would significantly increase the length of mapped tag sequences, permitting direct transferable marker assay development or higher resolution comparisons with related species for which better-defined genome sequence resources are available.

## Conclusions

Despite a relatively low proportion of markers overall (1.4 %) returning reliable orthologous matches to the *Prunus* genome sequence, the analyses performed still provided a total of 222 reference points between the genomes of the two species. These loci were distributed throughout the nine chromosomes of *Physocarpus* and the eight chromosomes of *Prunus* and thus permitted a meaningful comparison between these two genera. The study revealed a high level of macro-synteny between the two genomes, as has been demonstrated in comparisons of other Rosaceous genomes [3, 7, 8]. Seven *Physocarpus* chromosomes appear to be highly syntenic with their *Prunus* counterparts and the remaining two, LG4 and LG5, display evidence of fusion-fission events between two *Prunus* chromosomes each. Thus, the study presented here provides further evidence of a simple chromosomal rearrangement by which the derived *Prunus* genome evolved from a nine chromosome ancestral state to eight chromosomes. Analysis of the genomes of further Rosaceous genera with a base chromosome number of *x* = 9 will reveal whether the chromosomal configuration of *Physocarpus* likely represents that of the ancestral Rosaceous genome, or a derived state that has retained the ancestral chromosome number.

## Additional files

10.1186/s13104-016-2069-4 Orthology maps of each individual *Physocarpus opulifolius* linkage group detailing the relationships between each and the eight pseudomolecules of the *P. persica* genome sequence through the mapping of orthologous markers. *Physocarpus* linkage groups are shown on the left of the figure and genetic distances are given in cM, whilst *Prunus pseudomolecules* are given to the right and their physical distances are given in 100,000 bp intervals. Links between linkage groups and *pseudomolecules* indicate the positions of orthologous markers on the two genomes.
10.1186/s13104-016-2069-4 Positions of orthologous markers mapped in *Physocarpus*, the linkage group and genetic position of each marker, and their corresponding *Prunus* chromosome and physical position.

## Abbreviations

LG: linkage group
PC: *Prunus* chromosome
