# Supplementary material for: Genotyping-by-sequencing in an orphan plant species Physocarpus opulifolius helps identify the evolutionary origins of the genus Prunus
Source: BMC Res Notes. 2016 May 11;9:268. doi: 10.1186/s13104-016-2069-4 (PMC4864905; doi:10.1186/s13104-016-2069-4)
Supplement: Supplementary file 1 — 10.1186/s13104-016-2069-4 Orthology maps of each individual Physocarpus opulifolius linkage group detailing the relationships between each and the eight pseudomolecules of the P. persica genome sequence through the mapping of orthologous markers. Physocarpus linkage groups are shown on the left of the figure and genetic distances are given in cM, whilst Prunus pseudomolecules are given to the right and their physical distances are given in 100,000 bp intervals. Links between linkage groups and pseudomolecules indicate the positions of orthologous markers on the two genomes. [file 13104_2016_2069_MOESM1_ESM.docx]

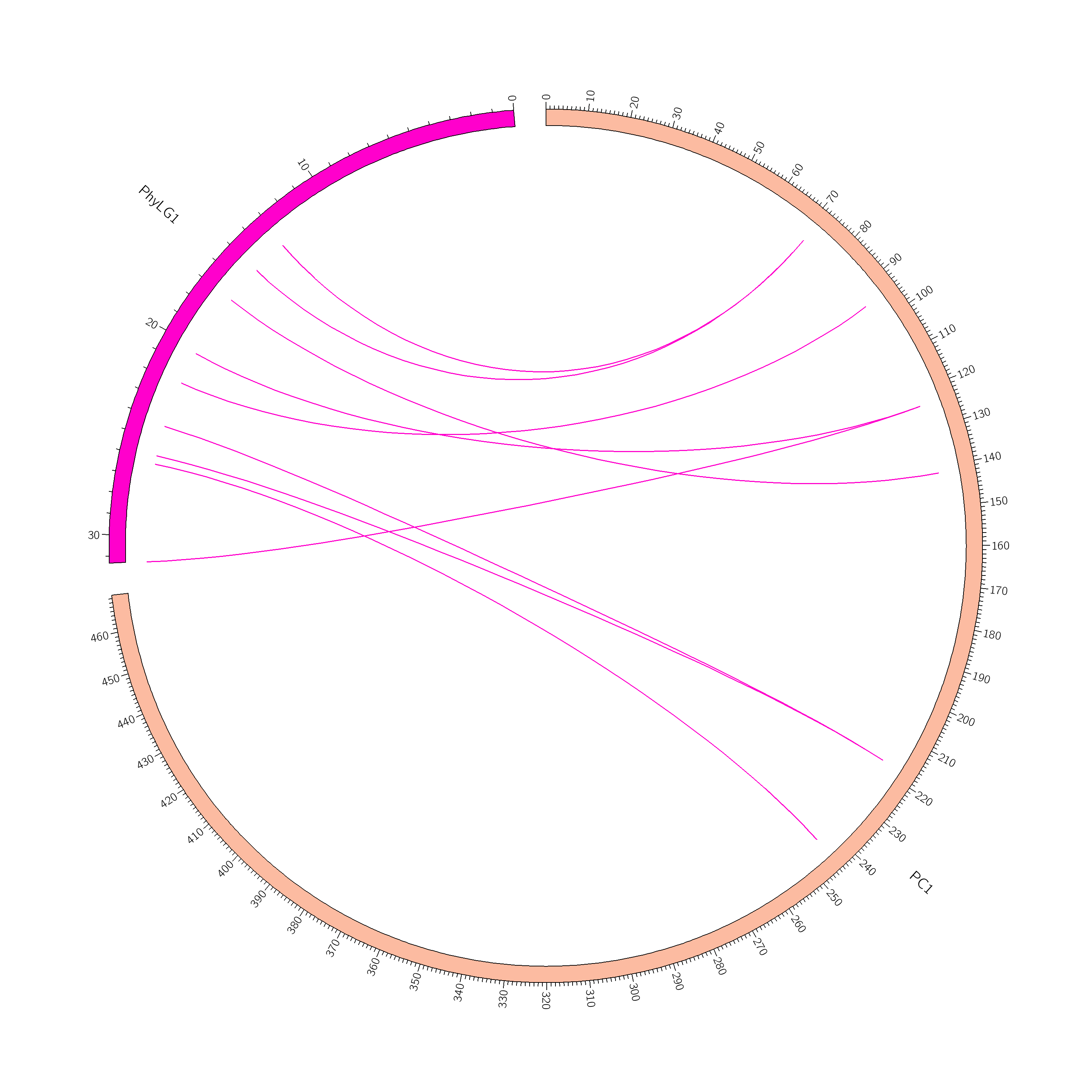


**Figure S1**. An orthology map of the relationships between *Physocarpus* linkage group LG1 and *Prunus* pseudomolecule PC1. *Physocarpus* genetic distances are given in cM, whilst *Prunus* physical distances are given in 100,000 bp intervals. Links indicate the positions of orthologous markers on the two genomes.


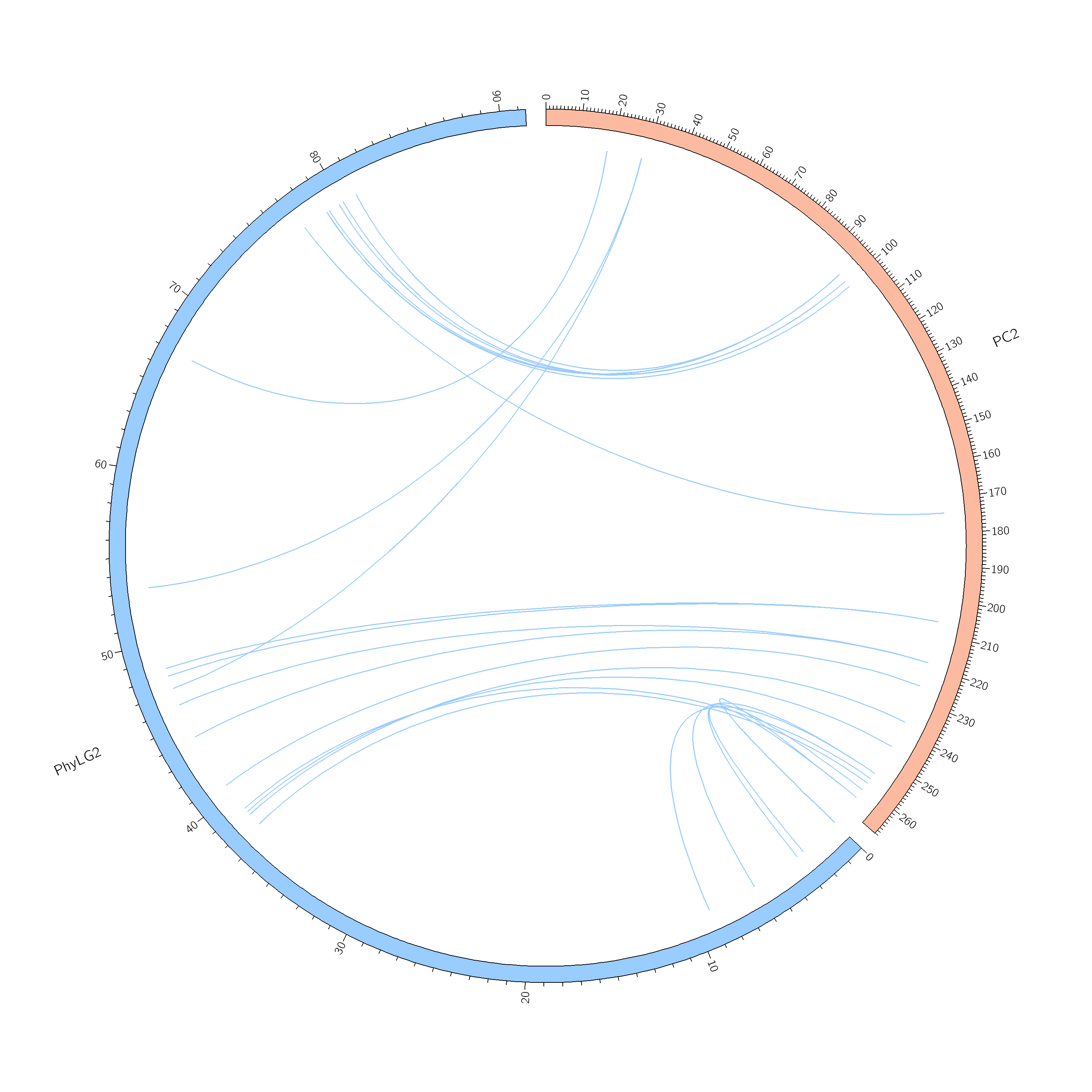


**Figure S2**. An orthology map of the relationships between *Physocarpus* linkage group LG2 and *Prunus* pseudomolecule PC2.


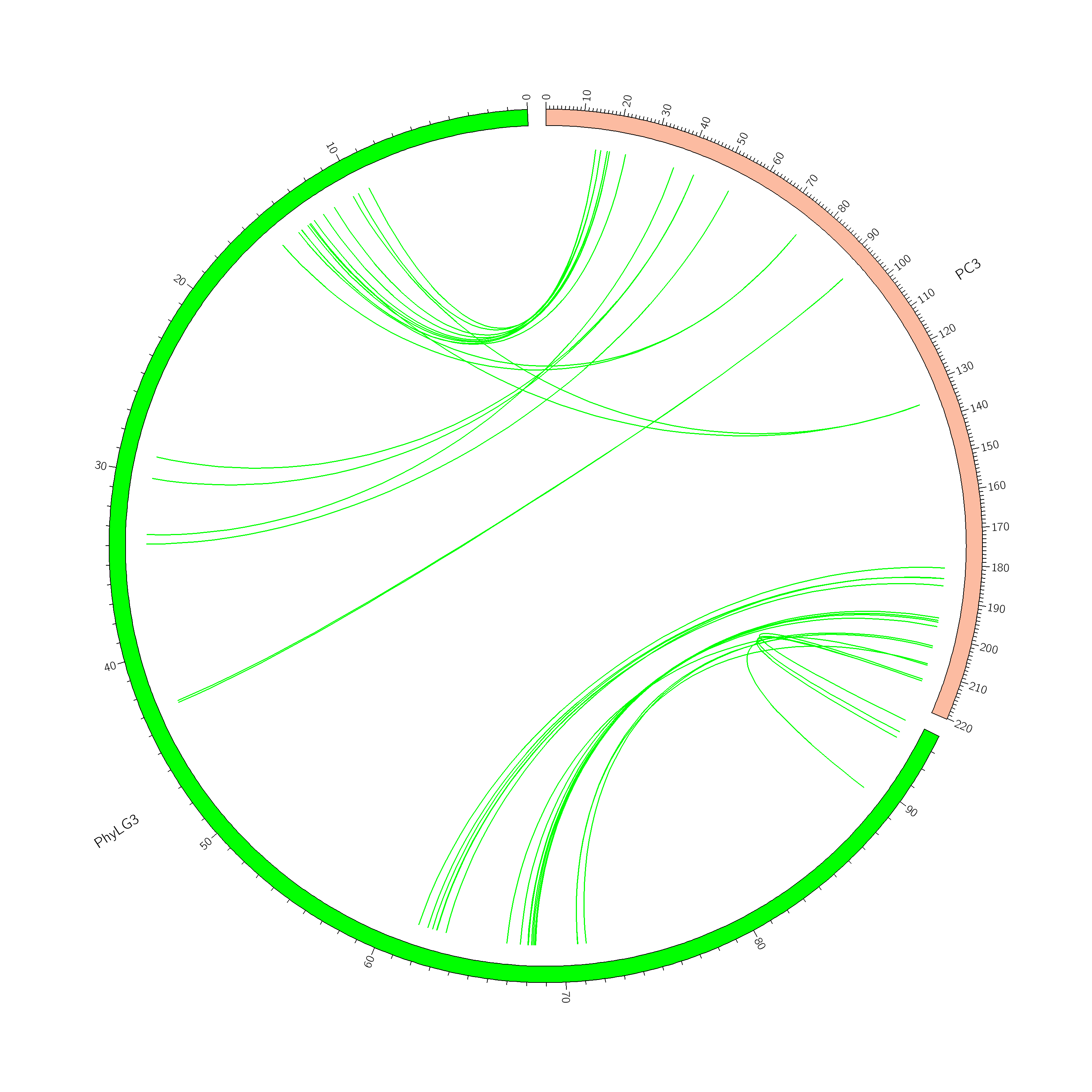


**Figure S3**. An orthology map of the relationships between *Physocarpus* linkage group LG3 and *Prunus* pseudomolecule PC3.


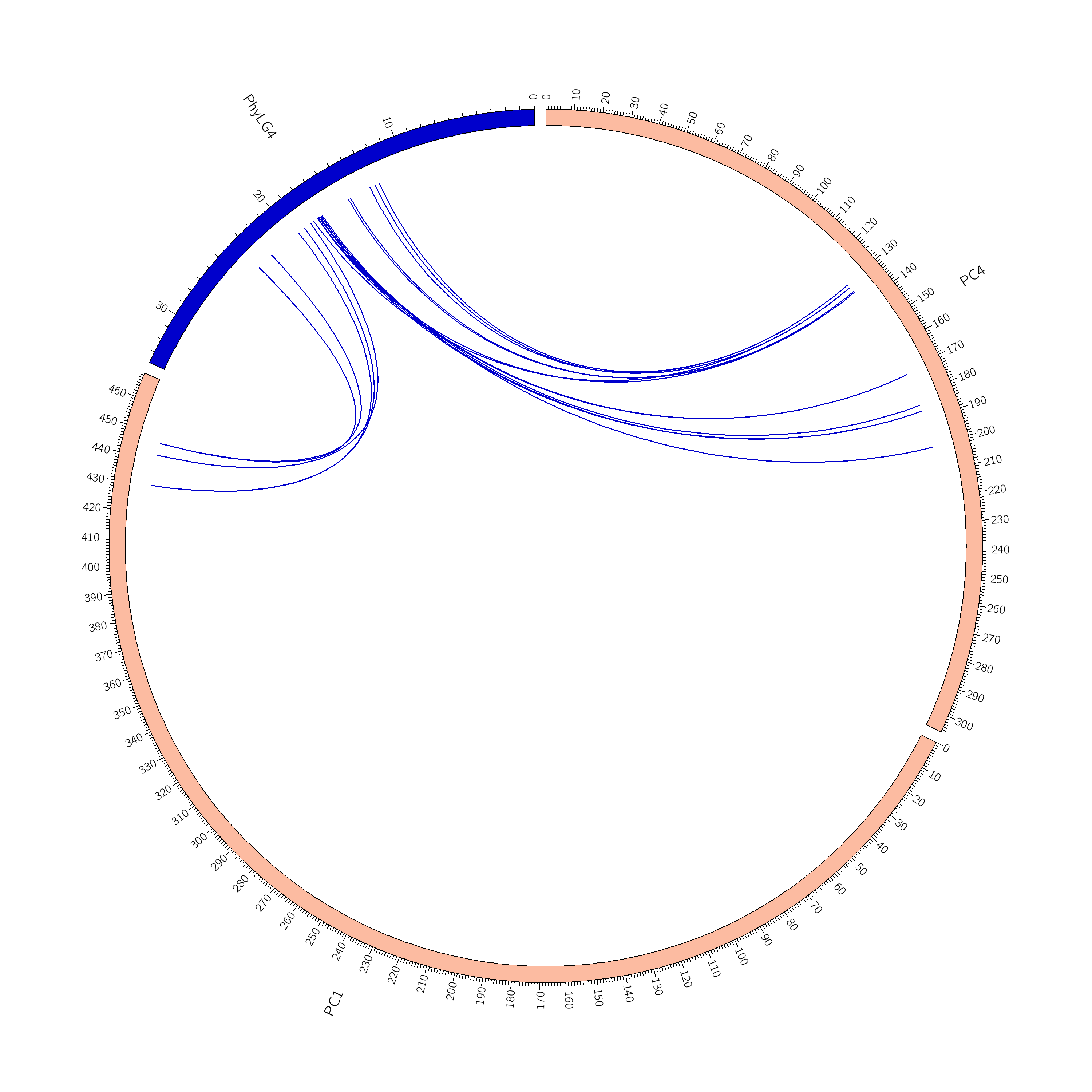


**Figure S4**. An orthology map of the relationships between *Physocarpus* linkage group LG4 and *Prunus* pseudomolecules PC4 and PC1.


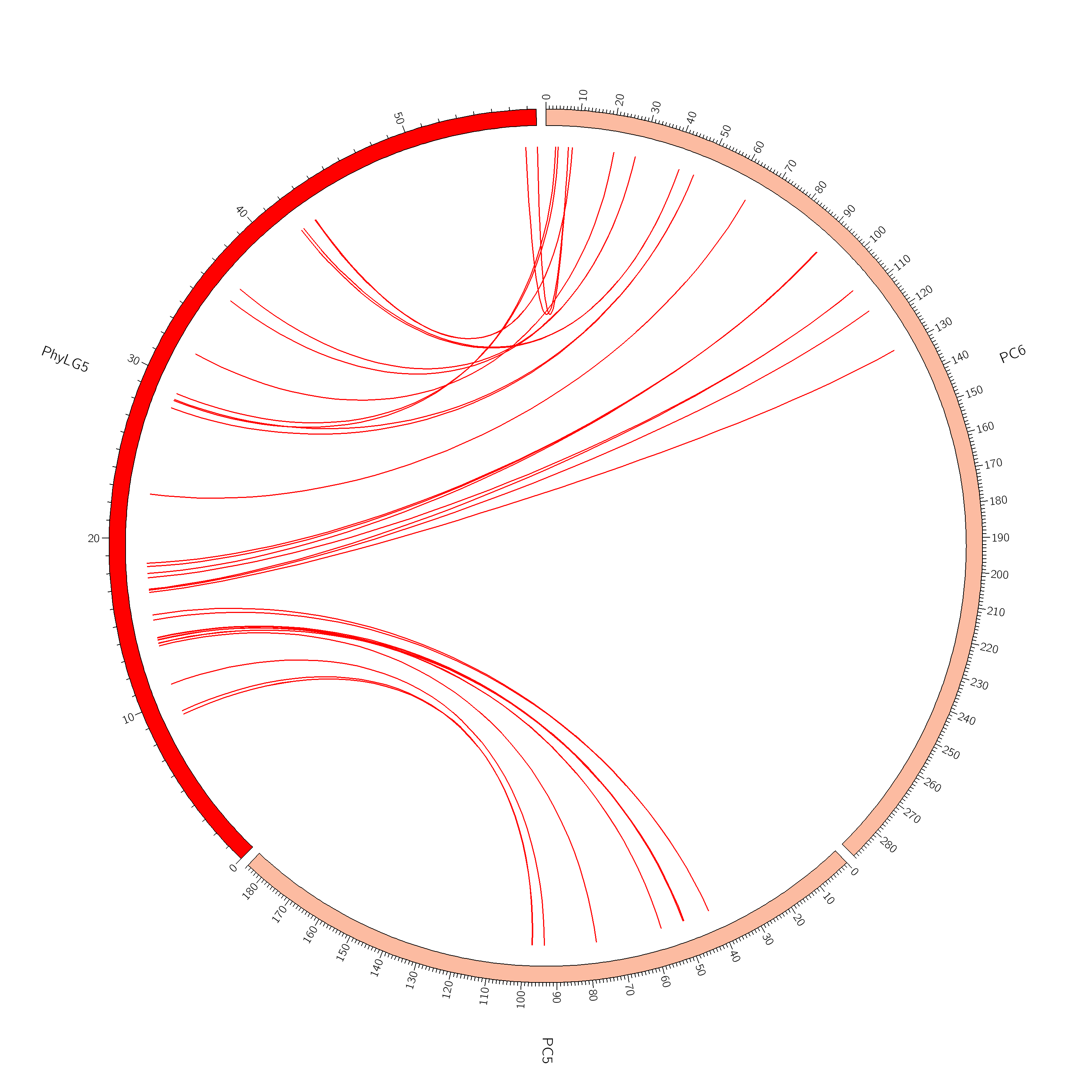


**Figure S5**. An orthology map of the relationships between *Physocarpus* linkage group LG5 and *Prunus* pseudomolecule PC5 and PC6.


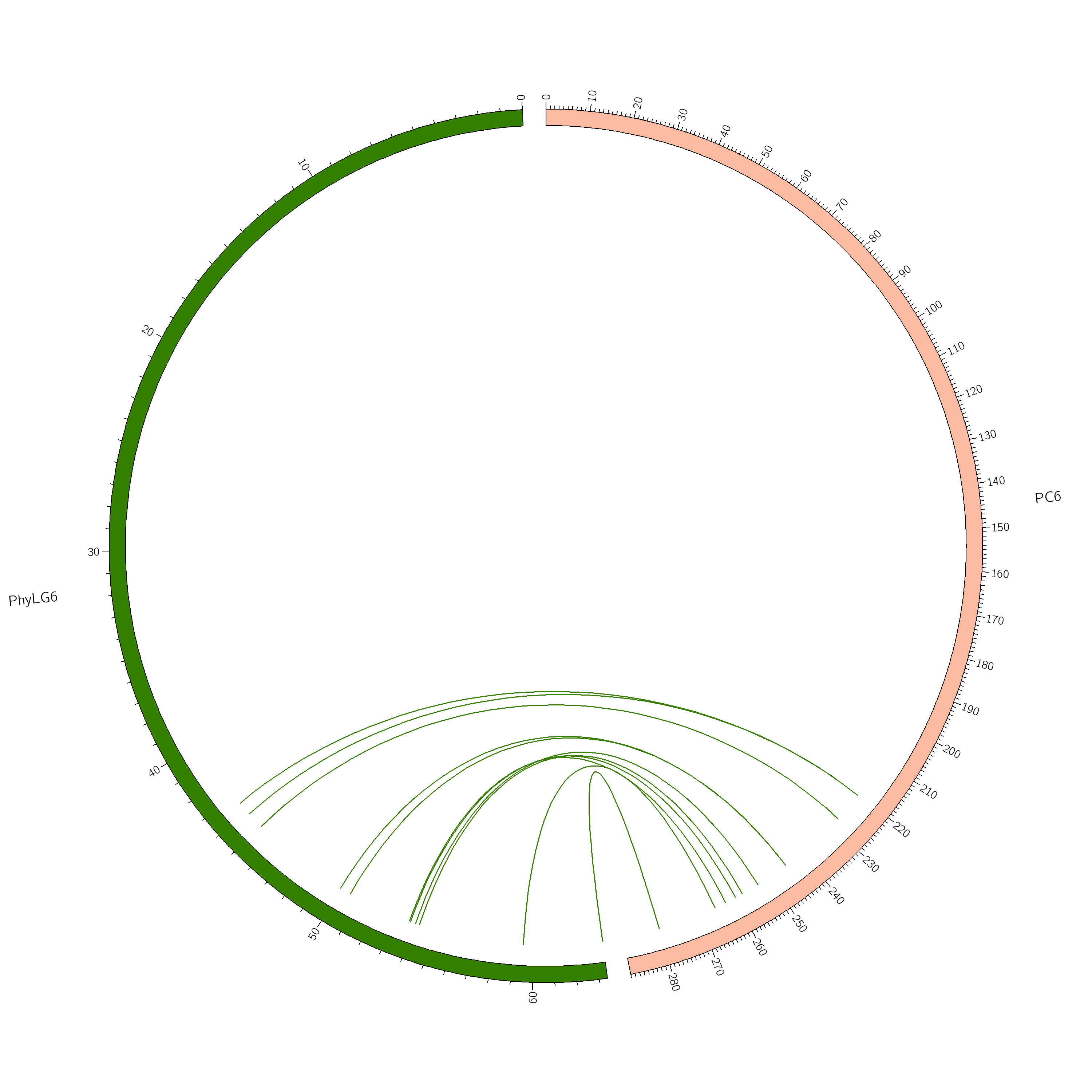


**Figure S6**. An orthology map of the relationships between *Physocarpus* linkage group LG6 and *Prunus* pseudomolecule PC6.


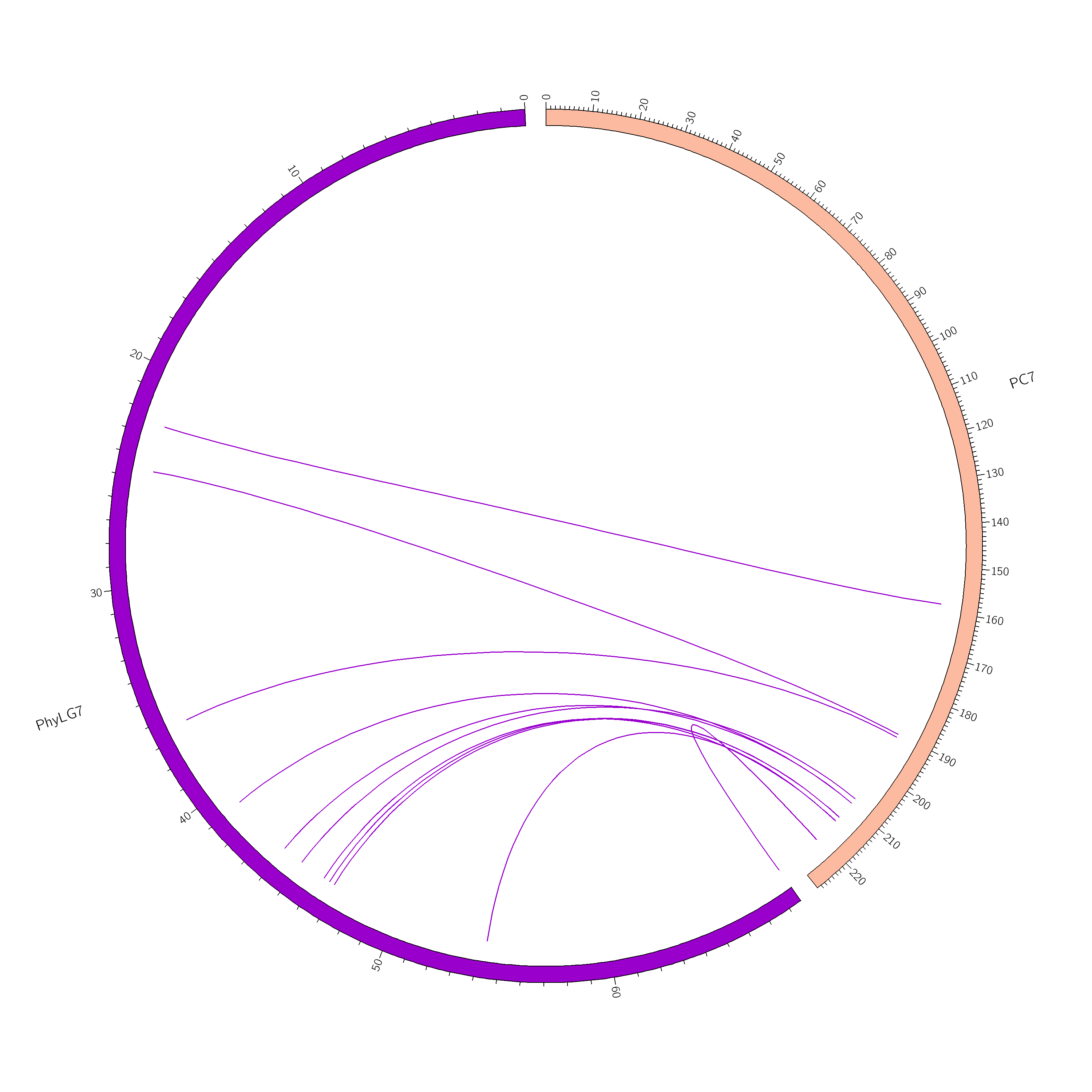


**Figure S7**. An orthology map of the relationships between *Physocarpus* linkage group LG7 and *Prunus* pseudomolecule PC7.


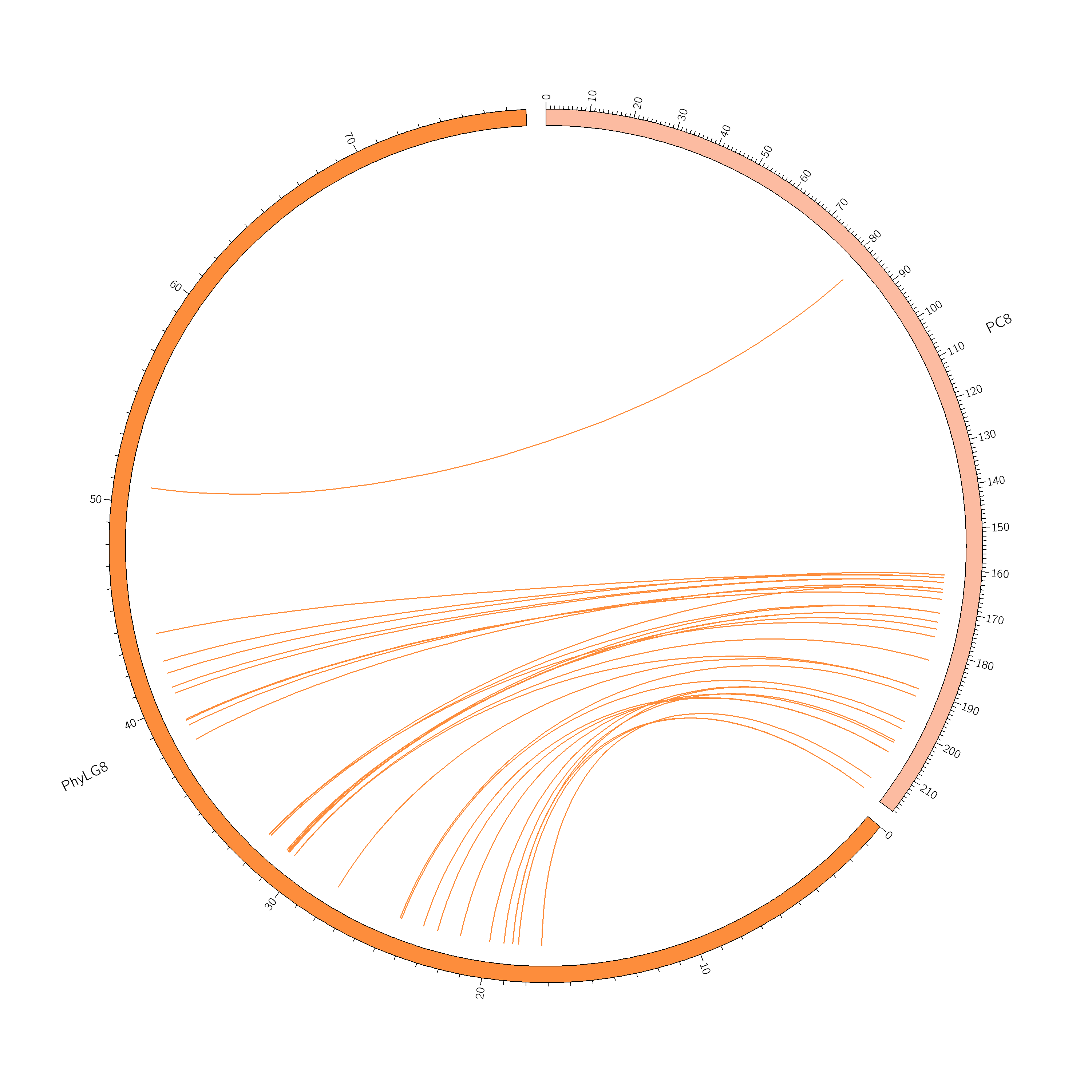


**Figure S8**. An orthology map of the relationships between *Physocarpus* linkage group LG8 and *Prunus* pseudomolecule PC8.


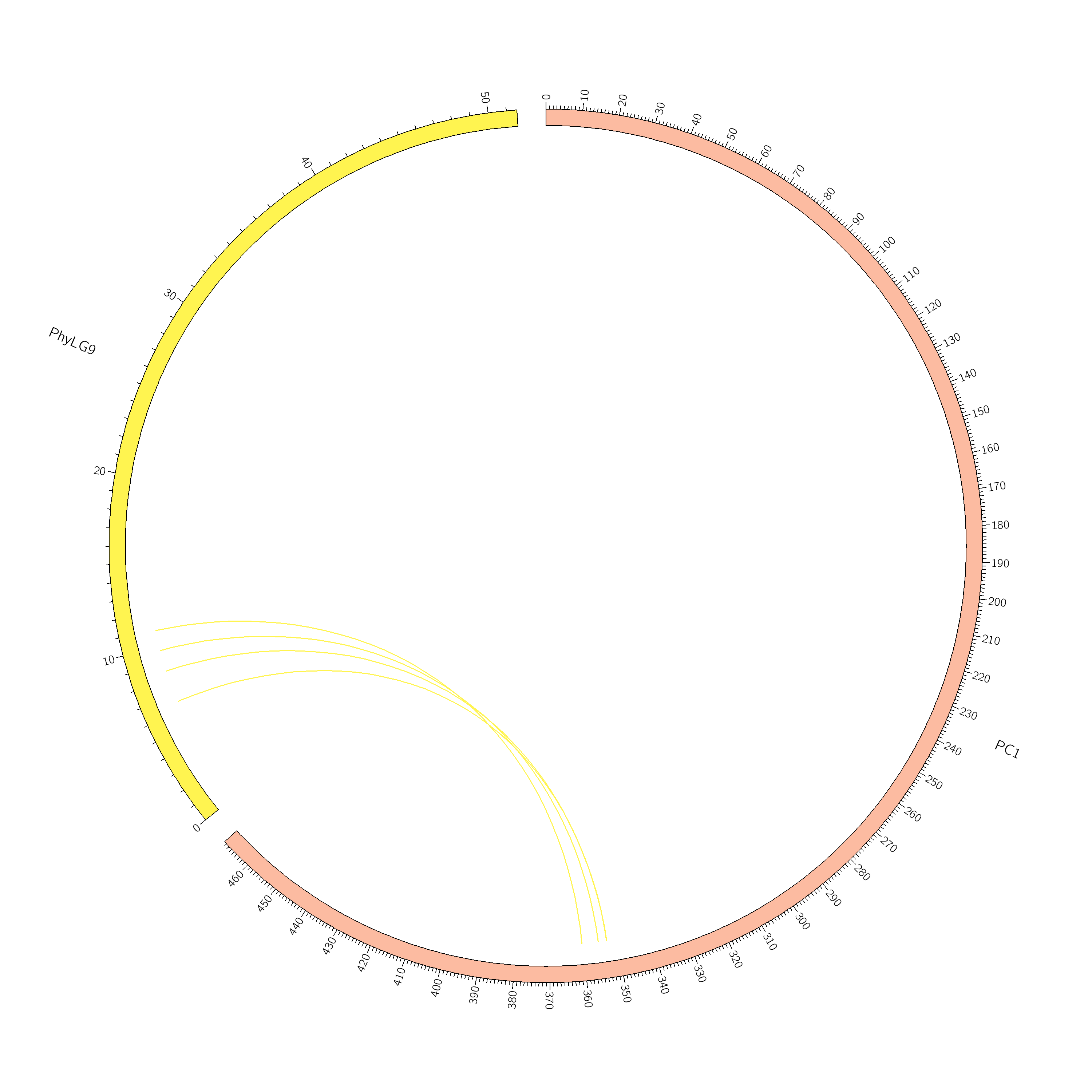


**Figure S9**. An orthology map of the relationships between *Physocarpus* linkage group LG9 and *Prunus* pseudomolecule PC1.
