# Supplementary material for: Genotyping-by-sequencing in an orphan plant species Physocarpus opulifolius helps identify the evolutionary origins of the genus Prunus
Source: BMC Res Notes. 2016 May 11;9:268. doi: 10.1186/s13104-016-2069-4 (PMC4864905; doi:10.1186/s13104-016-2069-4)
Supplement: Supplementary file 2 — 10.1186/s13104-016-2069-4 Positions of orthologous markers mapped in Physocarpus, the linkage group and genetic position of each marker, and their corresponding Prunus chromosome and physical position. [file 13104_2016_2069_MOESM2_ESM.pdf]

Marker name	*Physocarpus* linkage group	Genetic position	*Prunus* chromosome	Physical position
41423	PhyLG1	18.959	PC4	16526868
7914	PhyLG1	16.022	PC1	7138214
15679	PhyLG1	15.245	PC1	7138252
38959	PhyLG1	13.228	PC1	14138698
9830	PhyLG1	10.474	PC1	12367546
52307	PhyLG1	8.579	PC1	9467485
5462	PhyLG1	4.748	PC1	21785776
37974	PhyLG1	4.254	PC1	21785708
26738	PhyLG1	5.623	PC1	24422248
47505	PhyLG1	0	PC1	12366788
45853	PhyLG2	0	PC2	26371933
7956	PhyLG2	1.252	PC2	26372014
2393	PhyLG2	4.528	PC2	26085002
41831	PhyLG2	7.572	PC2	25487861
44357	PhyLG2	9.21	PC2	25487936
32792	PhyLG2	50.893	PC2	25840954
53581	PhyLG2	47.93	PC2	23739050
21472	PhyLG2	49.051	PC2	24554041
11988	PhyLG2	49.221	PC2	25681679
42380	PhyLG2	47.121	PC2	22589024
12834	PhyLG2	43.219	PC2	21868193
44185	PhyLG2	41.706	PC2	21868100
23646	PhyLG2	40.171	PC2	2828991
13742	PhyLG2	37.404	PC2	20633378
49233	PhyLG2	39.03	PC2	20633487
55551	PhyLG2	43.007	PC2	2828901
66748	PhyLG2	41.055	PC2	1793309
10216	PhyLG2	56.315	PC2	17428429
56801	PhyLG2	62.719	PC2	10112101
28843	PhyLG2	61.534	PC2	9661323
18725	PhyLG2	62.271	PC2	9661433
29748	PhyLG2	63.022	PC2	9918719
71147	PhyLG2	62.877	PC2	9918793
56035	PhyLG2	64.338	PC2	9660309
8281	PhyLG3	20.254	PC3	1392614
9387	PhyLG3	19.889	PC3	1392669
72522	PhyLG3	19.108	PC3	13462737
26507	PhyLG3	17.97	PC3	1533355
6796	PhyLG3	17.615	PC3	1712727
67971	PhyLG3	16.208	PC3	1533408
40543	PhyLG3	16.378	PC3	1774954
43644	PhyLG3	16.802	PC3	13462668
46232	PhyLG3	16.417	PC3	1712640
62525	PhyLG3	16.026	PC3	1774881

Marker name	*Physocarpus* linkage group	Genetic position	*Prunus* chromosome	Physical position
63334	PhyLG3	14.857	PC3	2230961
68096	PhyLG3	15.276	PC3	7528429
44099	PhyLG3	14.542	PC3	7528524
66056	PhyLG3	1.458	PC3	4211609
36252	PhyLG3	0	PC3	4211550
14632	PhyLG3	27.727	PC3	3617032
45789	PhyLG3	4.632	PC3	5285271
34405	PhyLG3	16.73	PC3	9326408
49258	PhyLG3	16.79	PC3	9326492
31136	PhyLG3	7.502	PC3	18092337
537	PhyLG3	8.041	PC3	18391961
45832	PhyLG3	8.314	PC3	18391873
68114	PhyLG3	8.571	PC3	18587524
54607	PhyLG3	8.873	PC3	18587595
30614	PhyLG3	9.01	PC3	18388800
32077	PhyLG3	12.273	PC3	20289879
58866	PhyLG3	14.601	PC3	19742156
34654	PhyLG3	13.378	PC3	19612304
29921	PhyLG3	13.578	PC3	19567181
66687	PhyLG3	13.697	PC3	19567269
55150	PhyLG3	13.702	PC3	19612373
46280	PhyLG3	13.77	PC3	19495151
58168	PhyLG3	13.822	PC3	19495242
39113	PhyLG3	16.334	PC3	20339668
49044	PhyLG3	16.558	PC3	20812556
53787	PhyLG3	16.72	PC3	20339761
72018	PhyLG3	33.942	PC3	20849420
66577	PhyLG3	38.459	PC3	21313088
19123	PhyLG3	38.461	PC3	21313032
51863	PhyLG3	39.207	PC3	21260638
44059	PhyLG4	17.176	PC4	13046120
35109	PhyLG4	16.698	PC4	13176138
41933	PhyLG4	16.909	PC4	13046009
55754	PhyLG4	18.786	PC4	13431733
49816	PhyLG4	17.527	PC4	13387655
56072	PhyLG4	15.467	PC8	11324105
56338	PhyLG4	16.102	PC8	11324209
22177	PhyLG4	16.068	PC4	20092020
14453	PhyLG4	17.307	PC4	17157723
38780	PhyLG4	17.297	PC4	18425716
21244	PhyLG4	17.329	PC4	17160932
1384	PhyLG4	17.395	PC4	18654103
18468	PhyLG4	18.487	PC4	13431824
41215	PhyLG4	17.8	PC4	18654173

Marker name	*Physocarpus* linkage group	Genetic position	*Prunus* chromosome	Physical position
64595	PhyLG4	19.185	PC4	13176198
39912	PhyLG4	14.502	PC1	44188370
70917	PhyLG4	17.984	PC1	43002712
14392	PhyLG4	16.051	PC1	43002747
42353	PhyLG4	20.791	PC1	44642628
51759	PhyLG4	13.378	PC1	44642488
49228	PhyLG5	7.419	PC7	20322596
41844	PhyLG5	8.728	PC5	9736150
54123	PhyLG5	8.67	PC5	9729231
21185	PhyLG5	8.966	PC5	9736052
50463	PhyLG5	10.814	PC5	9360563
9277	PhyLG5	13.436	PC5	7760755
45272	PhyLG5	13.255	PC5	5022848
58267	PhyLG5	13.415	PC5	5022843
61873	PhyLG5	13.56	PC5	4996314
45672	PhyLG5	13.655	PC5	5729307
61151	PhyLG5	13.738	PC5	5729412
59627	PhyLG5	13.78	PC5	5022774
65420	PhyLG5	14.827	PC5	4176850
52820	PhyLG5	15.169	PC5	4176777
51757	PhyLG5	16.844	PC6	12970540
54502	PhyLG5	18.394	PC6	11536053
66570	PhyLG5	16.532	PC6	10746037
38735	PhyLG5	16.694	PC6	10745947
23312	PhyLG5	17.406	PC6	9114964
4287	PhyLG5	17.887	PC6	9127320
31310	PhyLG5	18.081	PC6	9127387
28433	PhyLG5	21.9	PC6	6397763
40769	PhyLG5	28.199	PC6	4640928
43148	PhyLG5	28.253	PC6	297062
51167	PhyLG5	28.862	PC6	4641013
19878	PhyLG5	27.369	PC6	376108
28593	PhyLG5	31.244	PC6	2090332
39660	PhyLG5	34.963	PC6	2762141
70578	PhyLG5	35.884	PC6	2762191
68753	PhyLG5	41.063	PC6	4160097
7148	PhyLG5	41.24	PC6	4160158
19639	PhyLG5	42.106	PC6	813886
24259	PhyLG5	42.148	PC6	813814
25049	PhyLG5	56.131	PC6	690668
14439	PhyLG5	57.085	PC6	690604
37006	PhyLG6	21.827	PC3	13514639
36501	PhyLG6	20.34	PC7	12561245
32666	PhyLG6	26.862	PC7	14599704

Marker name	*Physocarpus* linkage group	Genetic position	*Prunus* chromosome	Physical position
13246	PhyLG6	30.685	PC7	14607019
71619	PhyLG6	42.006	PC6	22033662
6934	PhyLG6	40.58	PC6	22033709
4249	PhyLG6	39.707	PC6	22786039
24600	PhyLG6	26.439	PC6	24511645
35415	PhyLG6	27.375	PC6	24511572
58639	PhyLG6	30.878	PC6	26252652
72382	PhyLG6	30.949	PC6	25975291
27342	PhyLG6	30.545	PC6	25339188
11419	PhyLG6	28.561	PC6	25779948
35317	PhyLG6	37.809	PC6	26538245
35364	PhyLG6	24.094	PC6	27998269
42668	PhyLG7	24.648	PC6	15949523
44434	PhyLG7	37.527	PC7	15840290
47674	PhyLG7	44.763	PC7	19026526
25875	PhyLG7	31.722	PC7	19098008
51337	PhyLG7	22.868	PC7	20946263
60410	PhyLG7	22.956	PC7	20946347
59105	PhyLG7	29.699	PC7	20817313
62661	PhyLG7	16.164	PC5	9674848
3272	PhyLG7	19.633	PC7	21504986
32479	PhyLG7	20.704	PC7	21381577
8004	PhyLG7	20.891	PC7	21381674
56817	PhyLG7	23.321	PC4	4597320
33423	PhyLG7	25.129	PC7	21505069
23483	PhyLG7	0	PC7	22112421
44664	PhyLG8	15.466	PC8	21487102
2127	PhyLG8	15.409	PC8	21790831
3201	PhyLG8	15.867	PC8	21790900
47819	PhyLG8	14.328	PC8	20071031
48285	PhyLG8	16.385	PC8	20403638
68266	PhyLG8	13.941	PC8	20070972
25330	PhyLG8	44.731	PC8	20461370
48010	PhyLG8	47.744	PC8	20727702
49641	PhyLG8	47.758	PC8	20727637
27020	PhyLG8	36.305	PC8	19877912
52996	PhyLG8	11.941	PC8	19195122
15809	PhyLG8	40.836	PC8	19000794
57021	PhyLG8	40.724	PC8	19000706
18114	PhyLG8	8.012	PC8	18258867
51887	PhyLG8	2.311	PC8	17302578
52048	PhyLG8	5.13	PC8	17302649
32599	PhyLG8	6.034	PC8	17071846
67738	PhyLG8	5.897	PC8	17476276

Marker name	*Physocarpus* linkage group	Genetic position	*Prunus* chromosome	Physical position
32599	PhyLG8	6.034	PC8	17071846
46033	PhyLG8	32.016	PC8	17669251
30954	PhyLG8	4.014	PC8	16476203
57730	PhyLG8	3.279	PC8	16476310
72650	PhyLG8	5.505	PC8	16554079
49906	PhyLG8	32.553	PC8	16730058
9341	PhyLG8	32.739	PC8	16730114
30625	PhyLG8	33.213	PC8	16316151
22084	PhyLG8	33.08	PC8	16316089
72598	PhyLG8	33.589	PC8	16124978
11395	PhyLG8	34.386	PC8	16199381
63046	PhyLG8	31.044	PC8	16199301
5466	PhyLG8	22.09	PC8	8236097
22359	PhyLG8	27.216	PC1	45529874
15220	PhyLG9	6.585	PC1	35308526
1545	PhyLG9	8.509	PC1	35308452
14964	PhyLG9	9.771	PC1	35562742
57430	PhyLG9	10.989	PC1	36050536
